# *In vitro* pharmacological characterization of standard and new lysophosphatidic acid receptor antagonists using dynamic mass redistribution assay

**DOI:** 10.3389/fphar.2023.1267414

**Published:** 2023-11-14

**Authors:** C. Ruzza, M. Argentieri, F. Ferrari, E. Armani, M. Trevisani, G. Marchini, G. Calo’

**Affiliations:** ^1^ Department of Neuroscience and Rehabilitation, University of Ferrara, Ferrara, Italy; ^2^ LTTA Laboratory for Advanced Therapies, Technopole of Ferrara, Ferrara, Italy; ^3^ Chiesi Farmaceutici SpA, Parma, Italy; ^4^ Department of Pharmaceutical and Pharmacological Sciences, University of Padua, Padua, Italy

**Keywords:** lysophosphatidic acid, lysophosphatidic acid receptors, *in vitro* pharmacology, dynamic mass redistribution assay, calcium mobilization assay, receptor antagonists and inverse agonists

## Abstract

Lysophosphatidic acid (LPA) is a bioactive phospholipid that acts as an agonist of six G protein-coupled receptors named LPA receptors (LPA_1-6_). LPA elicits diverse intracellular events and modulates several biological functions, including cell proliferation, migration, and invasion. Overactivation of the LPA–LPA receptor system is reported to be involved in several pathologies, including cancer, neuropathic pain, fibrotic diseases, atherosclerosis, and type 2 diabetes. Thus, LPA receptor modulators may be clinically relevant in numerous diseases, making the identification and pharmacodynamic characterization of new LPA receptor ligands of strong interest. In the present work, label-free dynamic mass redistribution (DMR) assay has been used to evaluate the pharmacological activity of some LPA_1_ and LPA_2_ standard antagonists at the recombinant human LPA_1_ and LPA_2_ receptors. These results are compared to those obtained in parallel experiments with the calcium mobilization assay. Additionally, the same experimental protocol has been used for the pharmacological characterization of the new compound CHI. KI 16425, RO 6842262, and BMS-986020 behaved as LPA_1_ inverse agonists in DMR experiments and as LPA_1_ antagonists in calcium mobilization assays. Amgen compound 35 behaved as an LPA_2_ antagonist, while Merck compound 20 from WO2012028243 was detected as an LPA_2_ inverse agonist using the DMR test. Of note, for all the compounds, similar potency values were estimated by DMR and calcium assay. The new compound CHI was found to be an LPA_1_ inverse agonist, but with potency lower than that of the standard compounds. In conclusion, we have demonstrated that DMR assay can be successfully used to characterize LPA_1_ and LPA_2_ ligands. Compared to the classical calcium mobilization assay, DMR offers some advantages, in particular allowing the identification of inverse agonists. Finally, in the frame of this study, a new LPA_1_ inverse agonist has been identified.

## 1 Introduction

Lysophosphatidic acid (LPA) is a bioactive phospholipid mainly synthesized by the enzyme autotaxin from membrane phospholipids. LPA is present in all eukaryotic tissues and plasma and regulates several cellular functions, acting as an agonist of six G protein-coupled receptors (GPCR) named LPA receptors (LPA_1-6_) (Yung et al., 2014; Geraldo et al., 2021). LPA_1_ (previously known as ventricular zone gene 1) was the first LPA receptor identified (Hecht et al., 1996). In the following years, the other LPA receptors were identified based on LPA_1_ homology (Noguchi et al., 2003; Kotarsky et al., 2006; Lee et al., 2006; Pasternack et al., 2008; Yanagida et al., 2009). LPA receptors are type I, rhodopsin-like GPCRs, and each receptor couples to different G proteins (G_12/13_, G_q/11_, G_i/0_, and G_s_) and activates different intracellular signaling pathways (Yung et al., 2014; Geraldo et al., 2021). Specifically, both LPA_1_ and LPA_2_ receptors are reported to couple to G_q/11_, G_i/0_, and G_12/13_ proteins (An et al., 1998; Geraldo et al., 2021; Yung et al., 2014), and for the LPA_1_ receptor, the recruitment of β-arrestin 2 has also been demonstrated (Sun and Lin, 2008). Additionally, LPA directly binds and activates the intracellular peroxisome proliferator-activated receptor-gamma (PPARγ) (McIntyre et al., 2003). Thus, LPA, eliciting diverse intracellular events, modulates several biological functions, including cell proliferation, migration, and invasion, behaving as a lipid growth factor (Ishii et al., 2004; Gotoh et al., 2012). Additionally, LPA stimulates the production of cytokines and reactive oxygen species (Shao et al., 2018). Overactivation of the autotaxin–LPA–LPA receptor axis is reported to be involved in several pathologies, including cancer (Gotoh et al., 2012; Balijepalli et al., 2021; Lin et al., 2021), fibrotic diseases (Pradère et al., 2008; Tager et al., 2008; Castelino et al., 2011; Oikonomou et al., 2012), neuropathic pain (Kuwajima et al., 2018), atherosclerosis, psoriasis (Gaire et al., 2020a), and type 2 diabetes (Geraldo et al., 2021). Moreover, the activation of the LPA_1_ receptor stimulates microglia activation and neuroinflammation (Kwon et al., 2018; Gaire and Choi, 2021), and the blockage of LPA_1_ has been proposed as protective in pathologies such as cerebral ischemia (Gaire et al., 2019; Lee et al., 2020), ischemic stroke (Gaire et al., 2020b), and spinal cord injury (Santos-Nogueira et al., 2015). Because of the role of LPA in several pathological conditions, in the last 20 years, LPA receptors have garnered special interest in drug discovery. Several non-lipid LPA receptor agonists and, particularly notably, antagonists have been identified and pharmacologically characterized, as reviewed well by Liu et al. (2021). These efforts have led to some LPA_1_ receptor antagonists entering clinical development for the treatment of fibrotic diseases. In particular, SAR-100842 (Schaefer et al., 2009) was tested in clinical phase II for the management of diffuse cutaneous systemic sclerosis (NCT01651143); SAR-100842 was found to be well-tolerated, but improvement in the modified Rodnan skin thickness score was not statistically significant (Allanore et al., 2018). BMS-986020 has been evaluated in patients with idiopathic pulmonary fibrosis (NCT01766817). Although a slower rate of decline in forced vital capacity was detected in BMS-986020-treated patients compared with placebo, this study was interrupted because use of the compound was associated with elevation in hepatic enzymes and with three cases of cholecystitis (Palmer et al., 2018). This limitation seems to have been overcome by the second-generation LPA_1_ antagonist BMS-986278 (Gill et al., 2022; Sivaraman et al., 2022), which is now in clinical phase II for the treatment of idiopathic pulmonary fibrosis and progressive fibrotic interstitial lung disease (NCT04308681) (Corte et al., 2021). No clinical trials in cancer patients have been performed until now with LPA receptor ligands.

**FIGURE 1:**
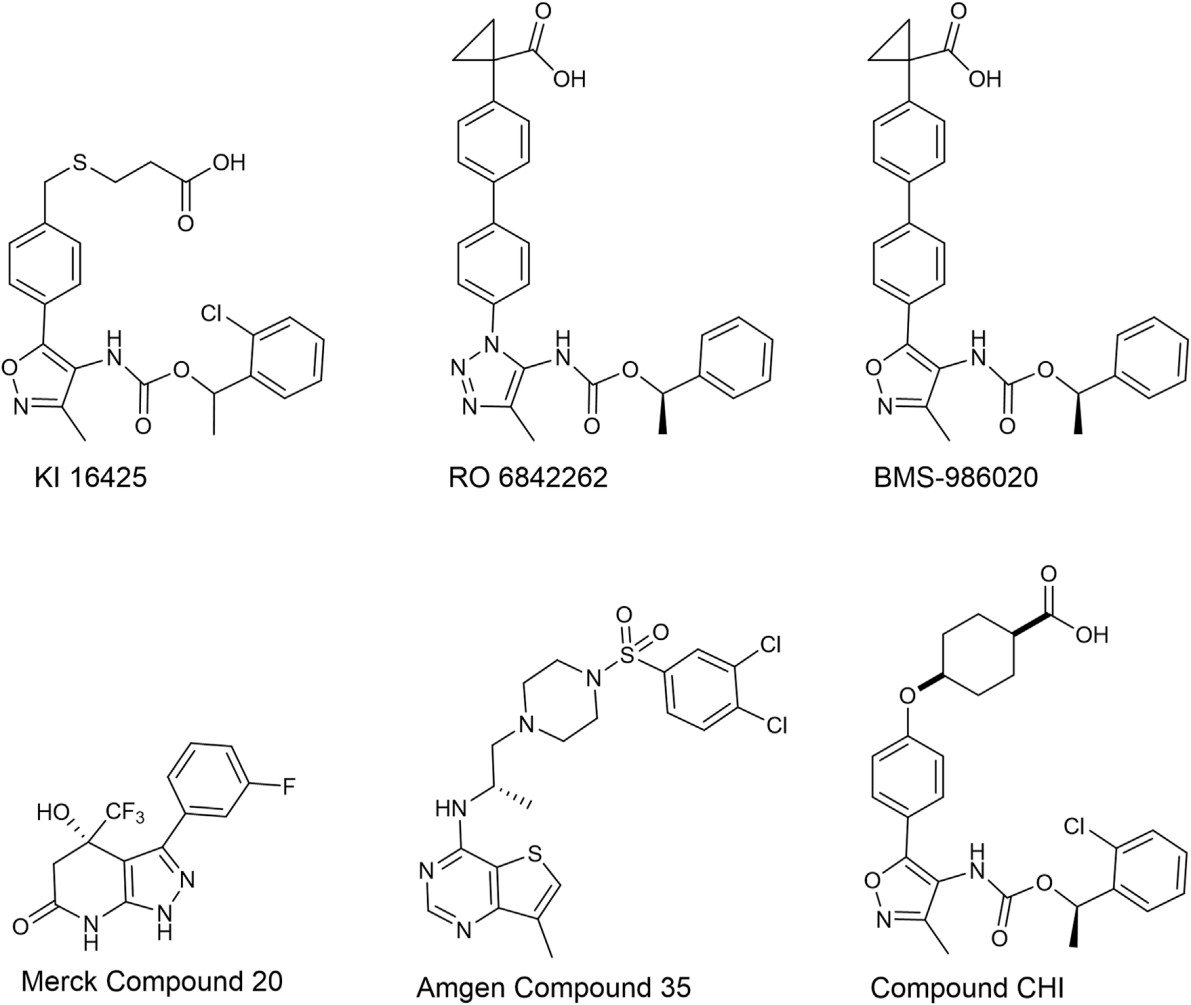
Chemical structures of the LPA receptor ligands used in this study.

Considering that LPA receptor modulators (i.e., antagonists, inverse agonists, and negative allosteric modulators) may be clinically relevant in numerous diseases, the identification and pharmacodynamic characterization of new LPA receptor ligands is of great interest. LPA ligands have been identified and studied using classical single endpoint assays (e.g., GTPγS binding) or distinct intracellular messenger levels (e.g., calcium mobilization). However, keeping in mind the complexity of the intracellular signaling pathways that follow LPA_1-6_ activation, the use of a whole-cell response assay can be advantageous. Dynamic mass redistribution (DMR) is a label-free assay that offers the possibility of obtaining, in a non-invasive manner, a holistic view of cellular responses after receptor activation. DMR uses an optical biosensor to translate the receptor-dependent holistic cellular response to a wavelength shift of an incident light in real time (Schröder et al., 2010; Grundmann and Kostenis, 2015). In the present research, the pharmacological profiles of three standard LPA_1_ and two standard LPA_2_ receptor antagonists have been investigated using DMR assays in CHO cells stably transfected with the LPA_1_ (CHO_LPA1_) and LPA_2_ (CHO_LPA2_) receptors. The results obtained are compared to those obtained in parallel experiments using the classical calcium mobilization assay. The standard LPA_1_ ligands used were KI 16425 (LPA_1/3_ antagonist, K_i_ estimated from binding experiments: 0.67 µM, Ohta et al., 2003), and the KI 16425 analogs RO 6842262 (Qian et al., 2012), and BMS-986020 (AKA BMS 986202 and AM152) (Cheng et al., 2021). The compound reported by Amgen as compound 35 in Beck et al. (2008) and the compound reported by Merck as compound 20 in Schiemann et al. (2012) were used as LPA_2_ antagonists. Additionally, a new LPA receptor ligand named compound CHI has been characterized in CHO_LPA1_ and CHO_LPA2_ cells using calcium mobilization and DMR assays. The chemical structures of all the compounds studied in the present work are shown in Figure 1.

## 2 Materials and methods

### 2.1 Drugs and reagents

KI 16425 was purchased from Aldrich; RO 6842262 was prepared as described in Gabriel et al. (2013); BMS-986020 was prepared as described in Hutchinson et al. (2010); and Amgen compound 35 and Merck compound 20 were prepared as described in the literature (Beck et al., 2008, Schiemann et al., 2012). Compound CHI was synthesized at Chiesi Laboratories. Oleoyl-L-α-lysophosphatidic acid sodium salt (LPA) was purchased from Merck KGaA (Darmstadt, Germany, product code L7260). Brilliant Black, bovine serum albumin (BSA), and 4-(2- hydroxyethyl)-1-piperazineethanesulfonic acid (HEPES) were obtained from Merck KGaA (Darmstadt, Germany). All cell culture media and supplements were purchased from Euroclone (Pero, Italy). LPA was dissolved in PBS 0.1% BSA (1 mM). LPA receptor antagonists were dissolved in dimethyl sulfoxide (DMSO, 10 mM stocks). Stock solutions were kept at –20°C until use. Serial dilutions were made up in assay buffer (Hanks’ Balanced Salt Solution (HBSS)/HEPES 20 mM, containing 0.01% BSA and 0.1% DMSO). For all LPA receptor antagonists, the highest concentration tested was 10 µM, with the exception of Compound 35, which, for solubility reasons, was used at the maximal concentration of 1 µM. Solvents, starting materials, and reagents for the preparation of CHI were purchased from Sigma-Aldrich or eNovation.

### 2.2 Cells

CHO_LPA1_ and CHO_LPA2_ cells were purchased from Eurofins DiscoverX. CHO cells were used as a control. Cells were maintained in Ham’s F12 medium supplemented with 10% FBS, 2 mM l-glutamine, 100 IU/mL penicillin, 100 IU/mL streptomycin, and 1 μg/mL Fungizone. Subsequently, 800 mg/mL G418 was added to the CHO_LPA1_ and CHO_LPA2_ cell media. Cells were cultured at 37°C in 5% CO_2_ humidified air.

### 2.3 Dynamic mass redistribution assay

Confluent cells were sub-cultured using trypsin/EDTA and used for experiments. Cells were seeded at a density of 20,000 cells/well in 30 µL into fibronectin-coated EnspireTM-LC 384-well plates and cultured for 20 h to form a confluent monolayer. On the day of the experiment, the cells were manually washed twice and maintained with assay buffer (HBSS with 20 mM HEPES, 0.01% BSA) for 90 min before DMR experiments. DMR was monitored in real time with a temporal resolution of 44 s throughout the assay. Experiments were performed at 37°C using an EnSight Multimode Plate Reader (PerkinElmer). The agonism protocol was as follows: before addition of the ligand, the DMR signal was measured for 5 min, and the average of the signals recorded during this period was set as the baseline (pm = 0). Compounds are added manually in a volume of 10 µL, and the triggered DMR signals were recorded for 60 min. The antagonism protocol was as follows: antagonists were added manually 25 min before reading the 5 min baseline. After baseline establishment (pm = 0), LPA was injected, and the DMR signal was recorded for 60 min. The antagonist properties of ligands were measured by assessing the concentration–response curve to LPA in the absence and in the presence of a fixed concentration of the compound. Responses were described in the form of picometer shift over time (seconds) following the subtraction of values from vehicle-treated wells. Maximum picometer (pm) modification (peak) and area under the curve (AUC) were used to determine the agonist response.

### 2.4 Calcium mobilization assay

Cells were seeded at a density of 50,000 cells/well in 100 μL into black, clear-bottom 96-well plates. The following day, cells were incubated with medium supplemented with 2.5 mM probenecid, 3 μM of the calcium-sensitive fluorescent dye Fluo-4 AM, and 0.01% pluronic acid for 30 min at 37°C. After the incubation period, the loading solution was aspirated, and 100 μL of HBSS supplemented with 20 mM HEPES, 2.5 mM probenecid, and 500 μM Brilliant Black was added. Serial dilutions of compounds were carried out in HBSS/HEPES (20 mM) buffer containing 0.01% BSA. Cell culture and drug plates were placed into a fluorimetric imaging plate reader (FlexStation II, Molecular Devices, Sunnyvale, CA), and fluorescence changes were measured. Online additions were carried out with a volume of 50 μL/well. The antagonist properties of ligands were measured by assessing the concentration–response curve to LPA in the absence and in the presence of a fixed concentration of the compound. Antagonists were injected into the wells 24 min before the addition of LPA. To facilitate drug diffusion into the wells, the experiments were performed at 37°C, and three cycles of mixing (25 μL from each well moved up and down three times) were performed immediately after injection of antagonists into the wells. Agonist effects were expressed in the form of maximum percentage change over baseline fluorescence. Baseline fluorescence was measured in wells treated with vehicle.

### 2.5 Data analysis and terminology

All data were analyzed using GraphPad Prism 9.4 (La Jolla, CA, United States). Concentration–response curves were fitted using the four-parameter log-logistic equation. Data are expressed in the form mean ± s.e.m. across *n* experiments performed in duplicate. Agonist potency is expressed in the form pEC_50_, which is the negative logarithm to base 10 of the agonist molar concentration that produces 50% of the maximal possible effect of that agonist. Antagonist potencies were assayed at single concentrations against the concentration–response curve to LPA. When antagonists did not change the LPA maximal effect, their pA_2_ was derived assuming a competitive type of antagonism, using the following equation: pA_2_ = log (CR-1)-log [B], where CR is the ratio between agonist potency (EC_50_) in the presence and agonist potency in the absence of antagonist and [B] is the molar concentration of the antagonist (Kenakin, 2004). When antagonists induced a significant reduction in LPA maximal effect, pK_B_ values were obtained by the Gaddum method (Gaddum et al., 1955). In practice, equiactive concentrations of the agonist in the absence ([A]) and presence ([A’]) of a non-competitive antagonist ([B]) were compared in a double reciprocal plot describing a straight line, and pK_B_ was derived from the equation: pK_B_ = log[(slope—1)/[B]]. The Gaddum method was applied for KI 16425 and RO 6842262, considering the concentration–response curve to LPA in the absence and in the presence of 0.1 µM of antagonist (calcium mobilization experiments, LPA_1_ receptor); for BMS-986020, considering the concentration–response curve to LPA in the absence and in the presence of 1 µM of antagonist (DMR experiments, LPA_1_ receptor); for compound 20, considering the concentration–response curve to LPA in the absence and in the presence of 1 µM of antagonist (calcium mobilization experiments, LPA_2_ receptor); for CHI, considering the concentration–response curve to LPA in the absence and in the presence of 1 µM of antagonist (calcium mobilization experiments, LPA_1_ receptor). Maximal effect data were analyzed using the Student’s t-test or a one-way ANOVA followed by Dunnett’s *post-hoc* test.

## 3 Results

### 3.1 LPA effects

**FIGURE 2:**
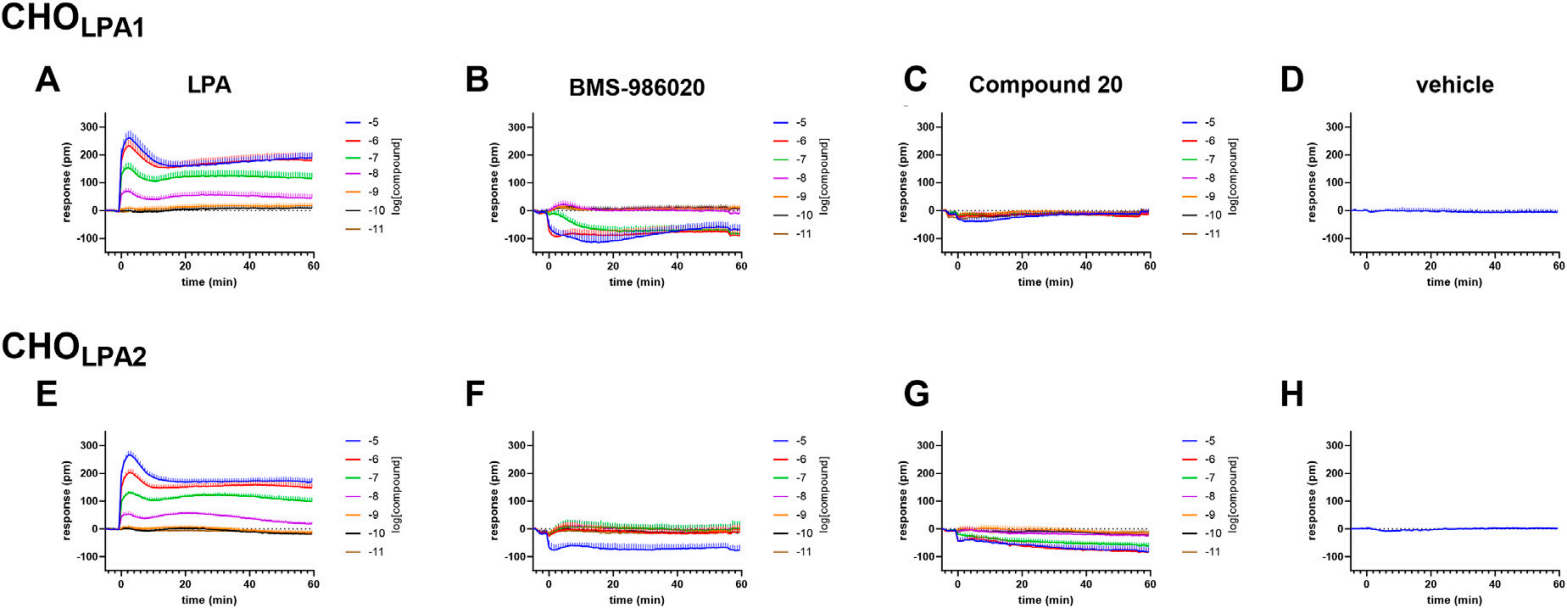
DMR experiments in CHO cells stably expressing the human LPA_1_ **(A–D)** or LPA_2_ **(E–H)** receptor. Averaged kinetics of increasing concentrations of LPA (A and E), BMS-986020 (B and F), and compound 20 (C and G). The DMR response elicited by the vehicle is shown in panels D and H. Data plotted represent the mean ± s.e.m across at least four independent experiments performed in duplicate.

**FIGURE 3:**
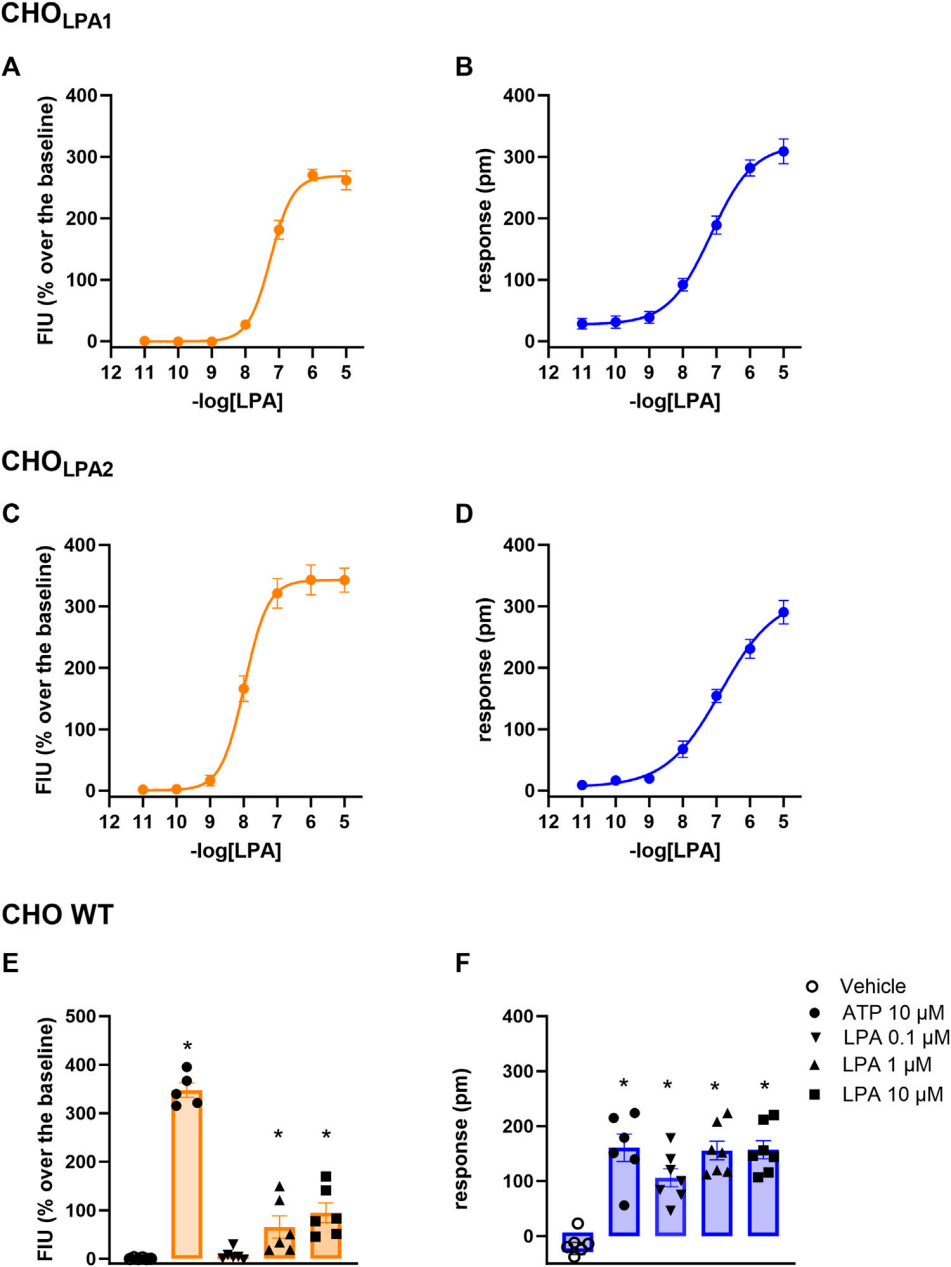
Concentration–response curve to LPA (0.01 nM–10 µM) in CHO_LPA1_ **(A, B)** and in CHO_LPA2_ **(C, D)** cells. Effects of 10–0.1 µM LPA and of 10 µM ATP in CHO wild-type cells **(E, F)**. Calcium mobilization experiment data are displayed in orange (panels A, C, and E), while blue plots show DMR data (panels B, D, and F). In DMR experiments, maximum picometer modifications (peaks) over a 60-min measurement period were used to generate concentration–response curves. Data plotted represent the mean ± s.e.m. across at least six independent experiments performed in duplicate. * *p* < 0.05 vs control, according to one-way ANOVA followed by Dunnet’s post *hoc test*.

In CHO_LPA1_ and CHO_LPA2_ cells, LPA produced stimulant effects in both calcium mobilization and DMR assay. Figure 2 shows the average DMR response elicited by increasing LPA concentrations over a 60-min measurement period in CHO_LPA1_ and CHO_LPA2_ cells. The LPA effects were computed in sigmoidal curves as peaks (Figure 3) and areas under the curve (AUC, Supplementary Material: S3), obtaining similar values for potency and efficacy. Of note, similar potency values were detected for both the receptors and in each of the two pharmacological assays. Specifically, in CHO_LPA1_ cells, pEC_50_ values of 7.25 (7.19–7.31) and 7.16 (6.89–7.43) were calculated for calcium mobilization and DMR assay, respectively. In CHO_LPA2_ cells, pEC_50_ values of 7.97 (7.89–8.05) and 6.87 (6.68–7.06) were calculated for the calcium mobilization and DMR assay, respectively. Importantly, at micromolar concentration, LPA also elicited some stimulant effects in CHO wild-type cells in both the assays (Figure 3). The pEC_50_ values estimated for LPA in this study are in line with other data reported in the literature, where LPA has exhibited a pEC_50_ of ∼7.5 at both recombinant (Swaney et al., 2011; Shimizu and Nakayama, 2017) and native (Sattikar et al., 2017) LPA_1_ receptor.

### 3.2 LPA_1_ receptor

**FIGURE 4:**
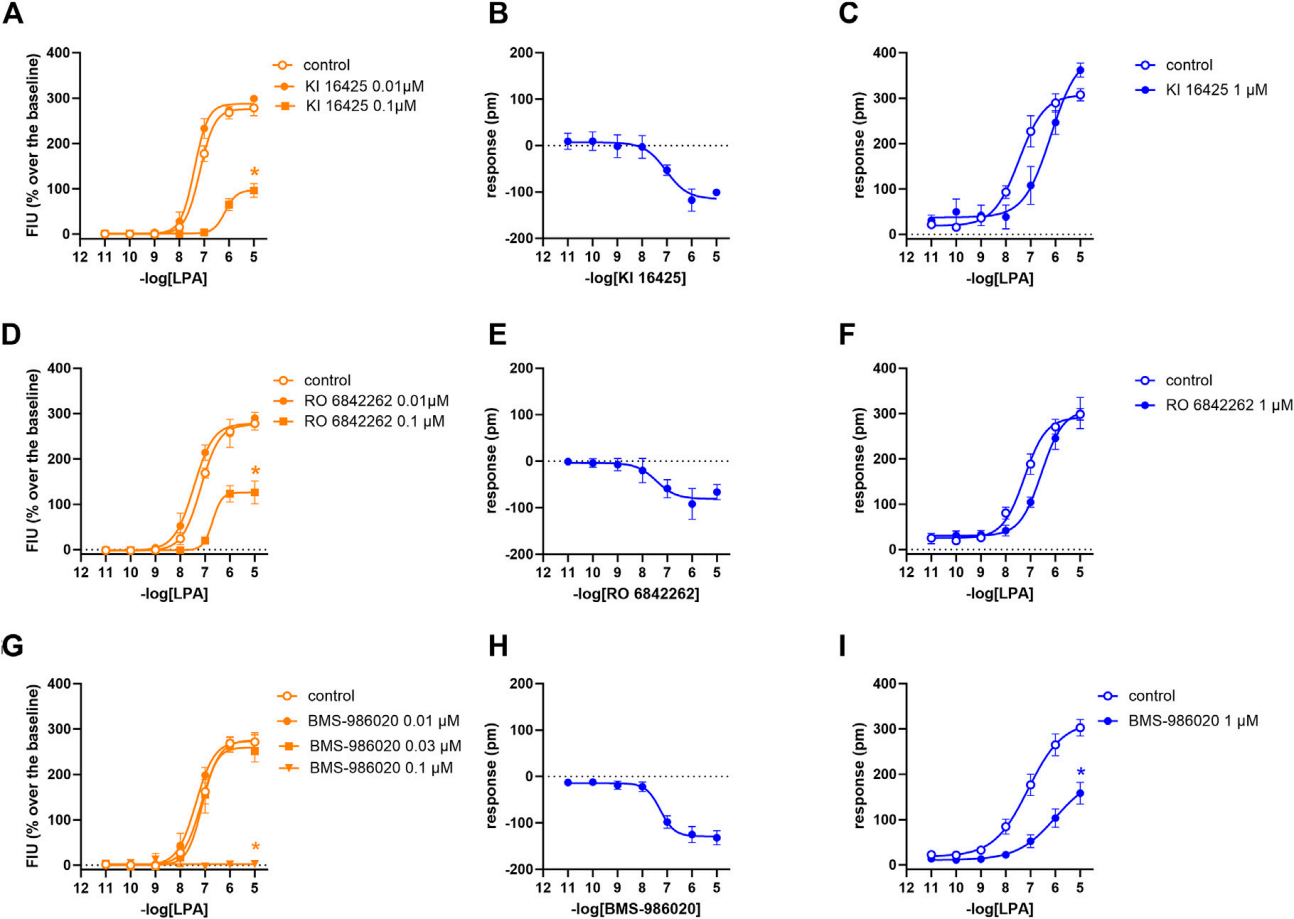
Pharmacological effects of KI 16425, RO 6842262, and BMS-986020 in CHO_LPA1_ cells. Calcium mobilization experiment data are displayed in orange **(A, D, G)**, while blue plots show DMR data **(B, C, E, F, H, I)**. Calcium mobilization experiments: concentration–response curves to LPA in the absence and in the presence of KI 16425 **(A)**, RO 6842262 **(D)**, and BMS-986020 **(G)**. DMR experiments: panels B, E, and H show the concentration–response curves to KI 16425, RO 6842262, and BMS-986020, respectively. Panels C, F, and I represent the concentration–response curves to LPA in the absence and in the presence of 1 µM KI 16425, RO 6842262, and BMS-986020, respectively. Data plotted represent the mean ± s.e.m. across at least three experiments performed in duplicate. *p < 0.05 vs. control, according to Student’s t-test **(C, F, I)** or one-way ANOVA followed by Dunnett’s *post hoc* test **(A, D, G)**.

**FIGURE 5:**
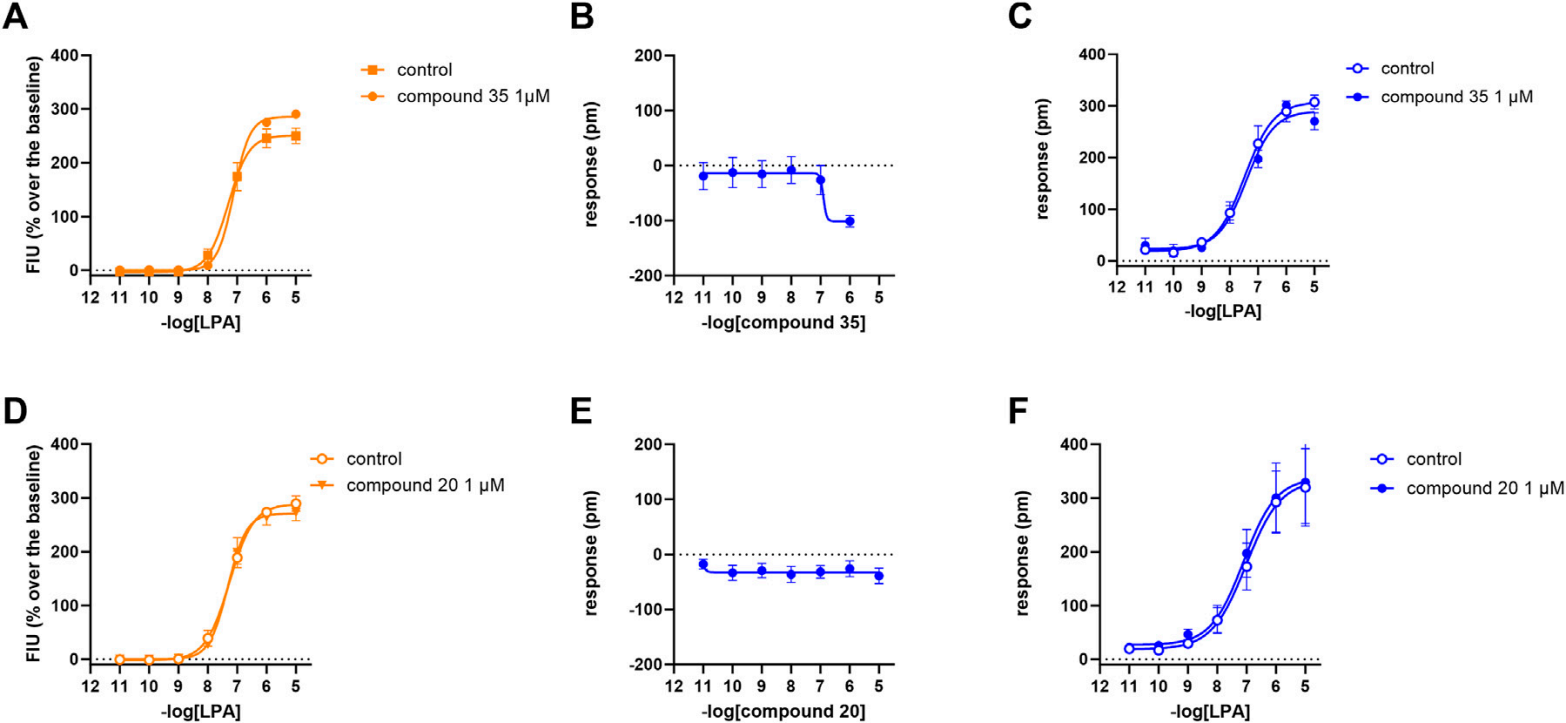
Pharmacological effects of compound 35 and compound 20 in CHO_LPA1_ cells. Calcium mobilization experiment data are displayed in orange **(A, D)**, while blue plots show DMR data **(B, C, E, F)**. Calcium mobilization experiments: concentration–response curves to LPA in the absence and in the presence of 1 µM compound 35 **(A)** and compound 20 **(D)**. DMR experiments: panels B and E show the concentration–response curves to compound 35 and compound 20, respectively. Panels C and F represent the concentration–response curves to LPA in the absence and in the presence of 1 µM compound 35 and compound 20, respectively. Data plotted represent the mean ± s.e.m. across at least three experiments performed in duplicate.

In the calcium mobilization assays performed in CHO_LPA1_ cells, KI 16425, RO 6842262, BMS-986020, Amgen compound 35, and Merck compound 20 did not produce any effect *per se* at 1 µM concentrations (data not shown). The same compounds were inactive at the 10 µM concentration in DMR experiments performed in CHO wild-type cells (Table 1 and Supplementary Material: S2). In contrast, when DMR assay was performed in CHO_LPA1_ cells, KI 16425, RO 6842262, and BMS-986020 produced concentration-dependent negative DMR signals, with different values of potency and efficacy (Figure 4, panels B, E, A). Specifically, RO 6842262 displayed higher potency, with pEC_50_ values close to 7.5. Lower potency was exhibited by KI 16425 and BMS-986020, with pEC_50_ values of 7.17 and 7.06, respectively (Table 2). In terms of efficacy, KI 16425 and BMS-986020 produced a negative DMR response of ∼ −110 pm, while RO 6842262 was less effective (∼-80 pm). In the case of DMR assay in CHO_LPA1_ cells, Merck compound 20 was inactive up to 10 μM, while Amgen compound 35 produced only a slight negative signal at the highest concentration tested (1 µM) (Figure 5, panels B and E). Figure 2 shows the average DMR response elicited by increasing concentrations of BMS-986020 and compound 20 over a 60-min measurement period, as illustrative compounds. The average DMR response elicited by increasing concentrations of KI 16425, RO 6842262, and compound 35 over a 60-min measurement period is shown in supplementary material (S1). Of note, the peak of the DMR response appeared in the first 20 min of the experiment for all the ligands, and no major differences were recorded in terms of potency or rank order of efficacy when the sigmoidal concentration–response curves were obtained using the AUC (supplementary material: S3) instead of peak effects. In CHO_LPA1_ cells, KI 16425, RO 6842262, and BMS-986020 shifted the concentration–response curve to LPA to the right, in the case of both calcium mobilization and DMR assay (Figure 4), with different pA_2_/pK_B_ values, as shown in Table 2. At 1 µM concentration, Amgen compound 35 and Merck compound 20 did not shift the concentration–response curve to LPA to the right in CHO_LPA1_ cells (Figure 5). Of note, KI 16425 and RO 6842262 reduced the LPA maximal effect in a concentration-dependent manner when tested as antagonists in calcium mobilization assay, but this pattern of activity was not replicated in DMR experiments. BMS-986020 produced a reduction in LPA maximal effect when tested both in calcium mobilization assay and in DMR experiments (Figure 4).

**TABLE 1:** DMR response (pm) evoked by the compounds in CHO cells.

	CHO	CHO_LPA1_	CHO_LPA2_
Vehicle	−24 ± 9	−2 ± 15	−16 ± 6
**KI 16425**	−42 ± 7	−101 ± 7*	−95 ± 4*
**RO 6842262**	−36 ± 12	−66 ± 16*	3 ± 21
**BMS-986020**	−39 ± 17	−134 ± 28*	−79 ± 18*
**Compound 20**	−26 ± 8	−31 ± 17	−119 ± 25*
**Compound 35**	−27 ± 13	−101 ± 10*	−88 ± 6*
**Compound CHI**	−4 ± 22	−78 ± 18*	−55 ± 19
**ATP**	156 ± 11*	84 ± 6	96 ± 13*

Data are expressed as mean DMR response (pm) ± s.e.m. across at least three experiments performed in duplicate. compound 35 1 μM, ATP 100 μM, other compounds 10 μM. * *p*< 0.05 vs vehicle, according to one-way ANOVA followed by Dunnet’s *post hoc* test. Vehicle: 0.001% DMSO in DMR buffer. Vehicle: 0.001% DMSO in DMR buffer.

**TABLE 2:** Pharmacological activity of LPA receptor standard ligands.

	CHO_LPA1_						CHO_LPA2_					
	Calcium mobilization			DMR			Calcium mobilization			DMR		
	Agonism		Antagonism	Agonism		Antagonism	Agonism		Antagonism	Agonism		Antagonism
	pEC_50_	E_max_	pK_B_/pA_2_	pEC_50_	E_max_	pK_B_/pA_2_	pEC_50_	E_max_	pK_B_/pA_2_	pEC_50_	E_max_	pA_2_
KI 16425	Inactive*		8.51 (7.94–9.08)	7.17 (5.60–8.74)	−110 ± 17	7.21 (6.73–7.69)	Inactive^#^		6.14 (5.25–7.03)	Crc incomplete		6.53 (5.27–7.79)
RO 6842262	Inactive*		8.69 (8.00–9.38)	7.31 (6.04–8.59)	−80 ± 16	6.54 (4.48–8.60)	Inactive^#^		<6	Inactive°		<6
BMS-986020	Inactive*		7 < pK_B_ < 7.5	7.06 (6.73–7.40)	−135 ± 13	7.10 (6.54–7.66)	Inactive^#^		6.76 (6.22–7.30)	Crc incomplete		<6
Compound 35	Inactive^#^		<6	Crc incomplete		<6	Inactive^#^		6.78 (5.97–7.59)	Crc incomplete		6.34 (5.05–7.63)
Compound 20	Inactive^#^		<6	Inactive°		<6	Inactive^#^		7.25 (6.90–7.60)	6.83 (6.40–7.25)	−94 ± 6	6.70 (5.85–7.55)

Data are expressed as mean ± s.e.m. across at least three experiments performed in duplicate. *inactive at 0.1 µM; #inactive at 1 μM; °inactive at 10 μM. pK_B_ for KI 16425 and RO 6842262 in calcium mobilization experiments at the LPA_1_ receptor, for BMS-986020 in DMR experiments at the LPA_1_ receptor, and for compound 20 in calcium mobilization experiments at the LPA_2_ receptor.

### 3.3 LPA_2_ receptor

**FIGURE 6:**
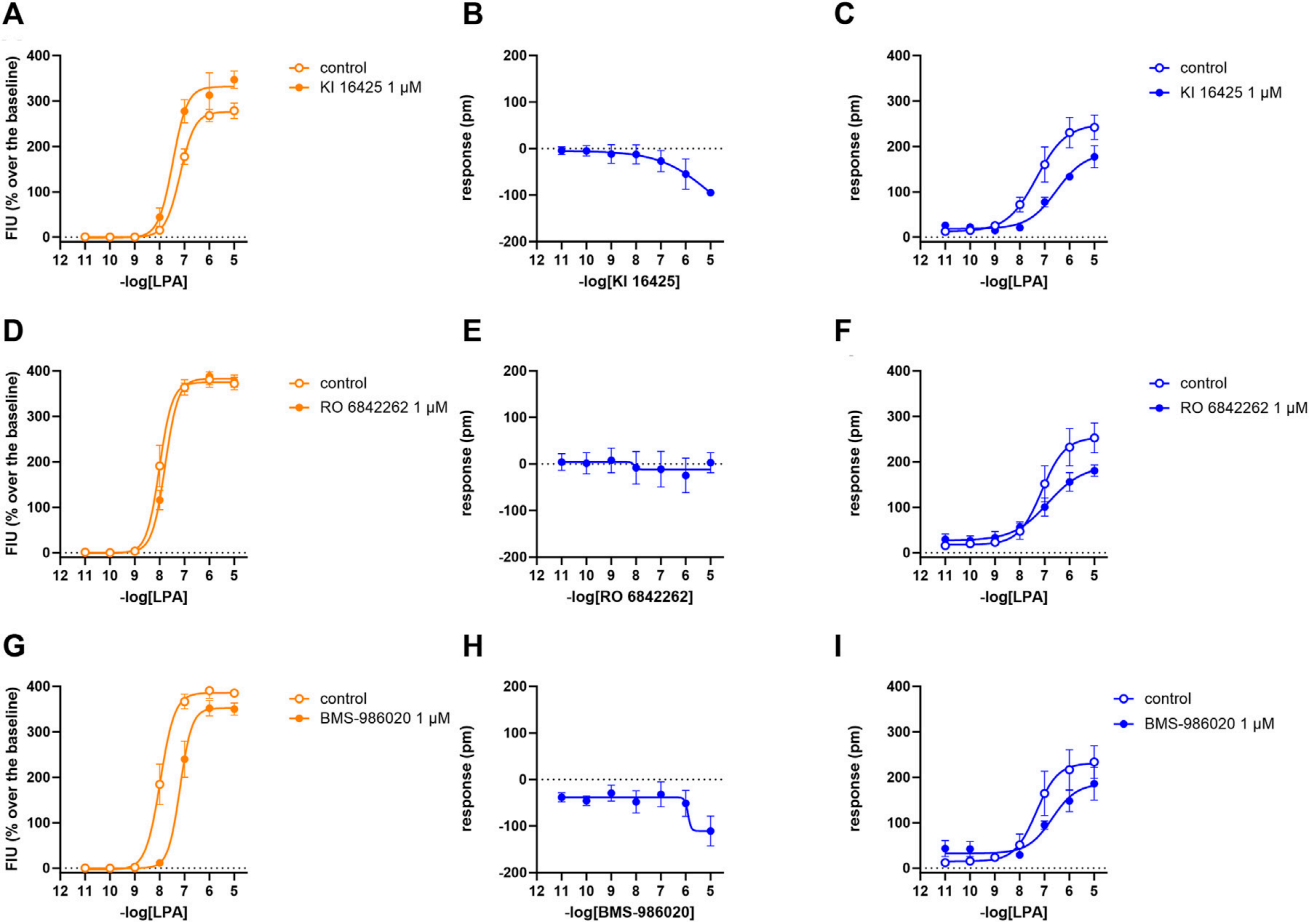
Pharmacological effects of KI 16425, RO 6842262, and BMS-986020 in CHO_LPA2_ cells. Calcium mobilization experiment data are displayed in orange **(A, D, G)**, while blue plots show DMR data **(B, C, E, F, H, I)**. Calcium mobilization experiments: concentration–response curves to LPA in the absence and in the presence of 1 µM KI 16425 **(A)**, RO 6842262 **(D)**, and BMS-986020 **(G)**. DMR experiments: panels B, E, and H show the concentration–response curves to KI 16425, RO 6842262, and BMS-986020, respectively. Panels C, F, and I represent the concentration–response curves to LPA in the absence and in the presence of 1 µM KI 16425, RO 6842262, and BMS-986020, respectively. Data plotted represent the mean ± s.e.m. across at least three experiments performed in duplicate.

**FIGURE 7:**
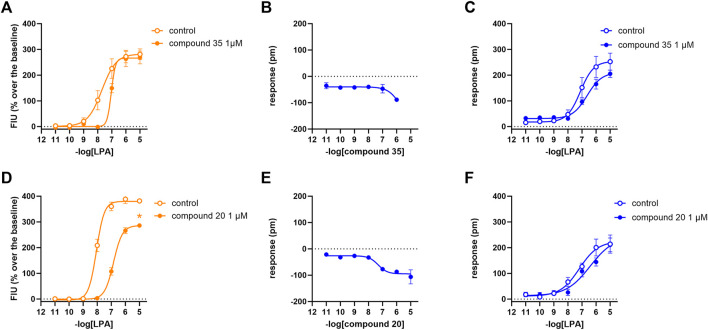
Pharmacological effects of compound 35 and compound 20 in CHO_LPA2_ cells. Calcium mobilization experiment data are displayed in orange **(A, D)**, while blue curves show DMR data **(B, C, E, F)**. Calcium mobilization experiments: concentration–response curves to LPA in the absence and in the presence of 1 µM compound 35 **(A)** and compound 20 **(D)**. DMR experiments: panels B and E show the concentration–response curves to compound 35 and compound 20, respectively. Panels C and F represent the concentration–response curves to LPA in the absence and in the presence of 1 µM compound 35 and compound 20, respectively. Data plotted represent the mean ± s.e.m. across at least three experiments performed in duplicate. *p < 0.05 vs. control, according to Student’s t-test.

In calcium mobilization assays performed in CHO_LPA2_ cells, KI 16425, RO 6842262, BMS-986020, Amgen compound 35, and Merck compound 20 did not produce any effect *per se* at 1 µM concentration (data not shown). Similarly, KI 16425, RO 6842262, BMS-986020 (Figure 6), and Amgen compound 35 (Figure 7) were inactive *per se*, or slightly active only at the higher concentration tested, in DMR assay. The only compound producing a negative DMR response in CHO_LPA2_ cells was Merck compound 20, with pEC_50_ and E_max_ of 6.83 and −94 pm, respectively (Figure 7). Of note, in this case, the maximum peak modification of the DMR response occurs ∼60 min after its administration; thus, this compound showed a slow onset of the effect compared to LPA and to other LPA ligands in CHO_LPA1_ cells (Figure 2). Regardless of this, similar concentration–response curves were obtained for this compound by fitting peaks (Figure 7) and the AUC (supplementary material: S3). Compound 20 (at 10 µM) failed to evoke effects *per se* in CHO wild-type cells (Table 1 and supplementary material: S2). When tested as antagonists, KI 16425, Amgen compound 35, and Merck compound 20 shifted the concentration–response curve to LPA to the right, both in calcium mobilization and in DMR assay, with different pA_2_/pK_B_ values, as shown in Table 2. BMS-986020 shifted the concentration–response curve to LPA to the right in CHO_LPA2_ cells only in the case of calcium mobilization assay, and not in DMR experiments. Furthermore, 1 μM RO 6842262 was not able to shift the concentration–response curve to LPA in these cells in the case of either assay (Figures 6 and 7).

### 3.4 Compound CHI

**FIGURE 8:**
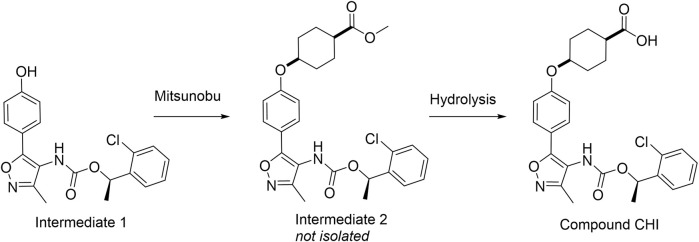
Scheme of CHI chemical synthesis.

Compound CHI was synthesized as depicted in Figure 8 via Mitsunobu and hydrolysis reactions starting from intermediate 1, prepared as described for intermediate 13.1 in Armani et al. (2022). 1H NMR spectra were recorded using 10 TMS as internal standard on Bruker Avance III HD 400 MHz or Bruker Fourier 300 MHz. Thin-layer chromatography was performed on Merck silica gel 60 F254 TLC plates or 60 RP-18 F254S TLC plates. Flash chromatography was performed on an Interchim PuriFlash 450 system. LCMS was performed on the Dionex UHPLC Ultimate 3000 apparatus with DAD 5 detector/Thermo Scientific MSQ Plus using a column Kinetex^®^ 2.6 pm XB-C18 (4.6. x 50 mm), 110 A, a mobile phase A: 0.1% formic acid in water and a mobile phase B: 0.1% formic acid in acetonitrile. HPLC conditions were as follows: wavelength range, 190–340 nm ±4 nm; flow, 1.0 mL/min; column temperature, 25°C; 10 elution gradient: time (min) mobile phase A (%) mobile phase B (%) flow (mL/min) 0.00 50 50 1.0 3.35 20 80 1.0 3.75 20 80 1.0 3.9 5 95 1.0 4.75 5 95 1.0 5.00 50 50 1.0 6.0 50 50 1.0. MS conditions were: mass range, 100–1,000 m/z; ionization, alternate; scan speed, 12,000 u/sec. Intermediate 1, (*R*)-1-(2-chlorophenyl)ethyl (5-(4-hydroxyphenyl)-3-methylisoxazol-4-yl)carbamate (0.1 g, 0.27 mmol, 1.0 eq), commercially available methyl (1*s*, 4*s*)-4-hydroxycyclohexane-1-carboxylate (0.13 g, 0.81 mmol, 3.0 eq), and diisopropyl azodicarboxylate DIAD (0.16 g, 0.81 mmol, 3.0 eq) were dissolved in anhydrous THF (10 mL) under argon atmosphere. Triphenylphosphine PPh_3_ (0.2 mL 0.081 mmol, 3.0 eq) was added, and the reaction mixture was stirred at RT overnight. The solvent was evaporated to dryness, and the crude was absorbed on SiO_2_ and purified via flash CC eluted by hexane: EtOAc (1:1, 0%–30% EtOAc in hexane). Fractions containing the main reaction product (intermediate 2, LCMS [M + H]^+^ = 513.2) were evaporated to dryness and dissolved in dioxane (5 mL) followed by the addition of 2M aqueous solution of LiOH (0.5 mL). The reaction mixture was stirred at room temperature overnight, neutralized by 1M HCl until pH < 4, and the solvent was evaporated to dryness. The crude was absorbed on SiO_2_ and purified via flash CC eluted by hexane: EtOAc (1:1, 0%–15% EtOAc in hexane). After evaporation to dryness of the fractions containing the main product, the title compound was obtained as a white solid (27 mg, 20% yield, purity 95%). ^1^H NMR (300 MHz, DMSO-*d*_6_) 12.16 (br. s., 1 H), 8.61–9.71 (m, 1 H), 7.60–7.90 (m, 2 H), 6.96–7.18 (m, 2 H), 6.74–7.62 (m, 4 H), 5.98 (q, J = 6.00 Hz, 1 H), 4.29–4.55 (m, 1 H), 2.25 (t, J = 11.05 Hz, 1 H), 2.08 (br. s., 3 H), 1.47–2.01 (m, 4 H), 1.02–2.13 (m, 4 H), 1.02–1.63 (m, 3 H). LCMS [M + H]^+^ = 499.2.

**FIGURE 9:**
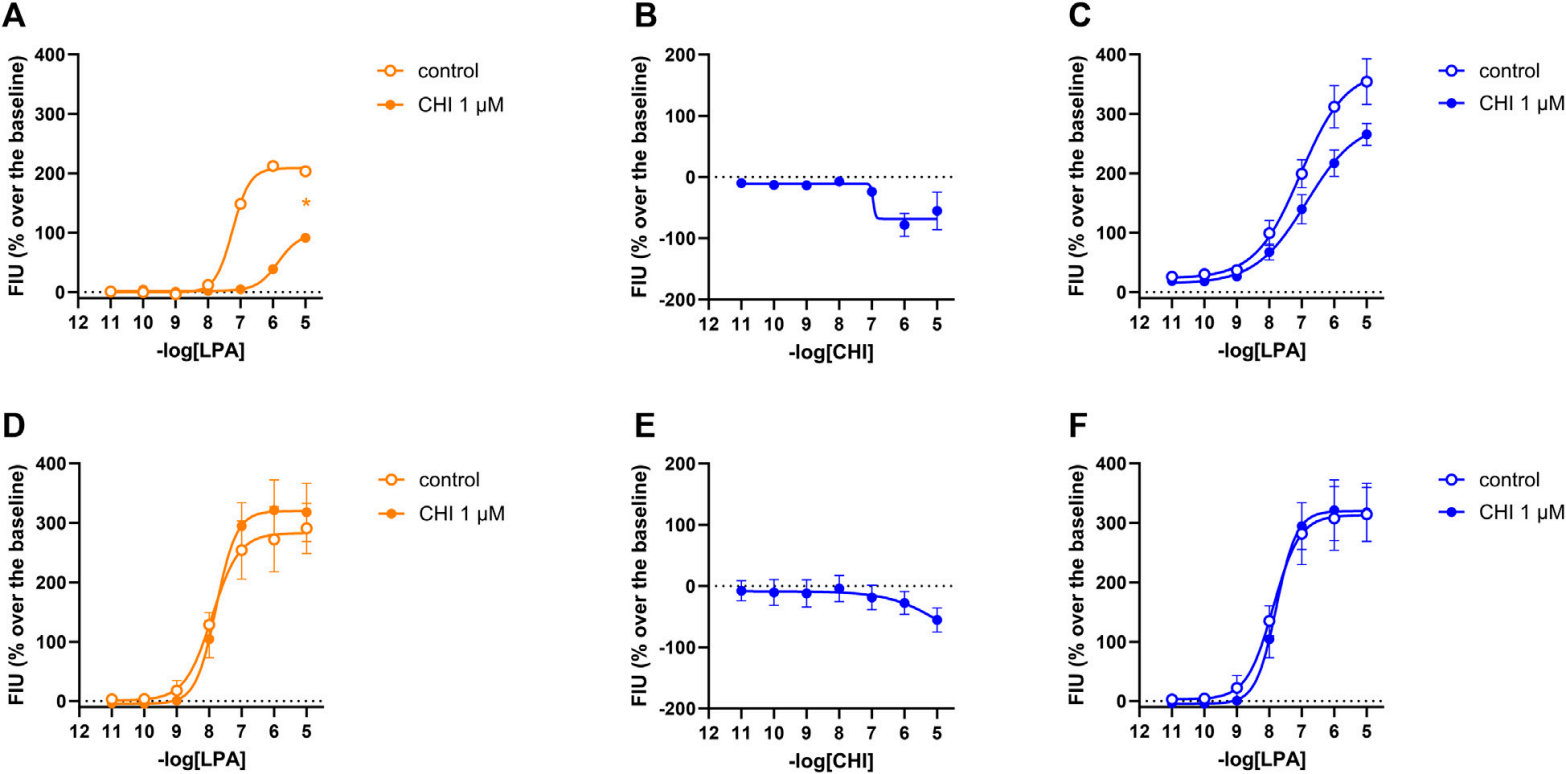
Pharmacological effects of compound CHI in CHO_LPA1_ (panels A, B, and C) and CHO_LPA2_ (panels D, E, and F) cells. Calcium mobilization experiment data are displayed in orange **(A, D)**, while blue plots show DMR data **(B, C, E, F)**. Calcium mobilization experiments: concentration–response curves to LPA in the absence and in the presence of 1 µM CHI in CHO_LPA1_ **(A)** and CHO_LPA2_ cells **(D)**. DMR experiments: panels B and E show the concentration–response curves to CHI in CHO_LPA1_ and CHO_LPA2_ cells, respectively. Panels C and F represent the concentration–response curves to LPA in the absence and in the presence of 1 µM CHI in CHO_LPA1_ and CHO_LPA2_ cells, respectively. Data plotted represent the mean ± s.e.m. across four experiments performed in duplicate. *p < 0.05 vs. control, according to Student’s t-test.

In calcium mobilization assays performed in CHO_LPA1_ and CHO_LPA2_ cells, compound CHI was found to be inactive at 1 µM (data not shown). In DMR experiments in CHO_LPA1_ cells, the compound produced a concentration-dependent negative DMR signal, with pEC_50_ and E_max_ of 6.51 (5.44–7.59) and −68 ± 13 pm, respectively. Under the same experimental conditions, compound CHI was inactive in CHO_LPA2_ and in CHO wild-type cells (Table 1 and supplementary material: S2). When tested as an antagonist at 1 µM concentration, compound CHI was active as an LPA_1_ antagonist only in calcium mobilization experiments (pK_B_ 7.24 (6.70–7.78)). Compound CHI did not shift the concentration–response curve to LPA in CHO_LPA2_ cells (Figure 9).

## 4 Discussion

The signaling of LPA receptors is complex and consists of various components (i.e., calcium mobilization, cAMP increase, and β-arrestin 2 recruitment). Despite this, for the screening and pharmacodynamic characterization of LPA ligands, assays quantifying only distinct second messengers such as cAMP or Ca^2+^, or the interaction of the receptor with β-arrestin, are commonly employed. Label-free technologies offer the opportunity to detect in real time the integrated cellular signal, thus providing a holistic unbiased view of all the intracellular events triggered by receptor activation. In the present work, the label-free assay DMR has been used to evaluate the pharmacological activity of a panel of LPA_1_ and LPA_2_ antagonists at the recombinant LPA_1_ and LPA_2_ receptors. These results have been systematically compared to those obtained in parallel experiments performed with calcium mobilization assay, a classical assay that is widely used to investigate LPA receptor ligands. Additionally, the same experimental protocols were used for the pharmacological characterization of the new compound CHI.

### 4.1 Antagonism

The compounds used as standard LPA_1_ and LPA_2_ antagonists were selected from among several LPA receptor ligands already published in the scientific and/or patent literature. The following compounds were selected as LPA_1_ antagonists: the isoxazole derivative KI 16425, first reported in 2001 by Ueno et al. (2003) and pharmacologically characterized *in vitro* in 2003 by Ohta et al. (2003); the N-aryltriazole derivative RO 6842262 (Gabriel et al., 2013); and BMS-986020 (Palmer et al., 2018). Among these, BMS-986020 has already entered clinical trials. In line with the findings reported in the literature, all the compounds were able to shift the concentration–response curve to LPA in LPA_1_ cells to the right, both in calcium mobilization and in DMR assay, thus behaving as LPA_1_ antagonists. In the present study, all the compounds exhibited similar potency values, close to 8 in calcium mobilization assay and ranging from 6.5 to 7.2 in the DMR test. Comparison of the potency values obtained in the present study with those reported in the literature is not always straightforward, possibly because of the different experimental approaches and conditions used. However, in general, no major differences between the results of the present study and those of previous studies have been noted. KI 16425 is one of the better characterized LPA_1/3_ antagonists. It was evaluated on the EDG-family LPA receptors (LPA_1_, LPA_2_, and LPA_3_) using GTPγS, inositol phosphate, and Ca^2+^ assays. KI 16425 has been reported to be a competitive LPA_1/3_ antagonist with pK_B_ values of ∼6.5/7 (Ohta et al., 2003), which is in line with the present results. BMS-986020 behaved as an LPA_1_ antagonist in calcium mobilization experiments, with pK_B_ close to 8 (Cheng et al., 2021). A small discrepancy between these data and those found in the scientific literature may be represented by RO 6842262, which has been reported to be 10-fold more potent than KI 16425 in inhibiting normal LPA-induced human lung fibroblast proliferation (Qian et al., 2012). This difference is likely to be attributable to the different kinds of biological preparations used and functions analyzed.

In terms of the type of antagonism exerted on the LPA_1_ receptor, in DMR experiments, KI 16425 and RO 6842262 shifted the concentration–response curve to LPA to the right without changing the maximal effect, thus suggesting a competitive type of antagonism. On the other hand, BMS-986020 shifted the concentration–response curve to LPA to the right along with a slight but statistically significant reduction in LPA maximal effect. Thus, a non-competitive/unsurmountable type of antagonism can be hypothesized; however, further studies using different compound concentrations are needed to better clarify this behavior. In contrast, in calcium mobilization experiments, all the LPA_1_ antagonists induced a strong reduction in LPA maximal effect, and for this reason, a precise pK_B_ value for BMS-986020 was not estimated. Moreover, it should be underlined that the pK_B_ values calculated for both KI 16425 and RO 6842262 via this assay using the Gaddum method were significantly higher (∼8.5) than those obtained in DMR experiments and those reported in the literature (∼7). For accurate interpretation of the behavior of KI 16425, RO 6842262, and BMS-986020 vs. the LPA concentration–response curve in calcium mobilization experiments, the fact that the rapid and transient nature of calcium peaks causes hemi-equilibrium conditions, especially when the antagonist slowly dissociates from the receptor, should be taken into account. This may lead to the appearance of unsurmountable behavior in competitive antagonists (Charlton and Vauquelin, 2010) and eventually to overestimation of their potency. Thus, we propose that the behavior of these LPA receptor antagonists in calcium mobilization assay is likely due to the features of the assay rather than to an unsurmountable type of antagonism, and that the pK_B_ values extrapolated for these compounds may be biased by this methodological issue. This hypothesis is supported by previous findings showing that KI 16425 induces a strong reduction in LPA maximal effects in calcium mobilization experiments but behaves as a competitive LPA_1/3_ antagonist in stimulated GTPγS binding and inositol phosphate accumulation assays (Ohta et al., 2003; Shimizu and Nakayama, 2017). DMR assay allows the system to reach equilibrium and offers a more precise instrument than calcium mobilization assay for qualitative definition of the type of antagonism exerted. Of note, similar differences between calcium mobilization and DMR have previously been reported for other antagonists and GPCRs, namely, nor-binaltorphimine at the kappa receptor (Sturaro et al., 2022) and [^*t*^Bu-D-Gly^5^]NPS at the neuropeptide S receptor (Ruzza et al., 2012; Ruzza et al., 2018). Importantly, the use in the present study of a single antagonist concentration strongly limits our ability to firmly define the nature of the antagonism exerted. Further studies using different concentrations of compounds vs. the concentration–response curve to LPA and the application of conventional Schild analysis will enable a firm classification of ligand behavior in terms of competitive vs. non-competitive antagonism. Merck compound 20 from Schiemann et al. (2012) and Amgen compound 35 (Beck et al., 2008) have been investigated as LPA_2_ receptor antagonists. In cells transfected with the LPA_2_ receptor, Amgen compound 35 has been found to inhibit the stimulant activity of LPA on Ca^2+^ mobilization with pIC_50_ of 7.77. In the same study, it was found to be highly selective for LPA_2_ vs. LPA_1_ and LPA_3_ receptors (Beck et al., 2008). Similarly, in cells expressing the LPA_2_ receptor, Merck compound 20 has been found to inhibit the effects of LPA on Ca^2+^ mobilization with pIC_50_ > 6 (Schiemann et al., 2012). In the present study, both Amgen compound 35 and Merck compound 20 behaved as LPA_2_ antagonists. Merck compound 20 was found to be slightly more potent than Amgen compound 35. Merck compound 20 induced a reduction in LPA maximal effects in calcium mobilization assay, likely due to the assay features mentioned previously.

The data collected in this work allow the evaluation of the LPA_1_ vs. LPA_2_ selectivity of the compounds. However, it is difficult to draw firm conclusions about ligand selectivity. In fact, at micromolar concentration, LPA was able to induce stimulant effects in CHO wild-type cells, both in calcium mobilization and in DMR assay. This suggests the presence of a certain number of natively expressed LPA receptors in CHO cells. Of note, these receptors may contribute to the effects elicited by LPA at high concentrations, which cannot be attributed solely to the recombinant proteins. Clearly, this is a relevant caveat that may lead to underestimation of the antagonist selectivity of action. Interestingly, a previous paper has reported on the expression of LPA receptors in CHO cells; using RT-PCR, the presence of LPA_1_ (but not LPA_2_ or LPA_3_) mRNA has been reported (Holdsworth et al., 2005). In the present study, KI 16425 and BMS-986020 behaved as both LPA_1_ and LPA_2_ antagonists, exhibiting only ∼10-fold selectivity for LPA_1_ (in calcium experiments). For KI 16425, this confirms the findings of a previous study reporting 20-fold selectivity for LPA_1/3_ vs. LPA_2_ (Ohta et al., 2003). RO 6842262 showed LPA_1_ selectivity >30-fold. As expected, Merck compound 20 and Amgen compound 35 were found to be LPA_2_, but not LPA_1_, antagonists at the concentration used.

### 4.2 Inverse agonism

**FIGURE 10:**
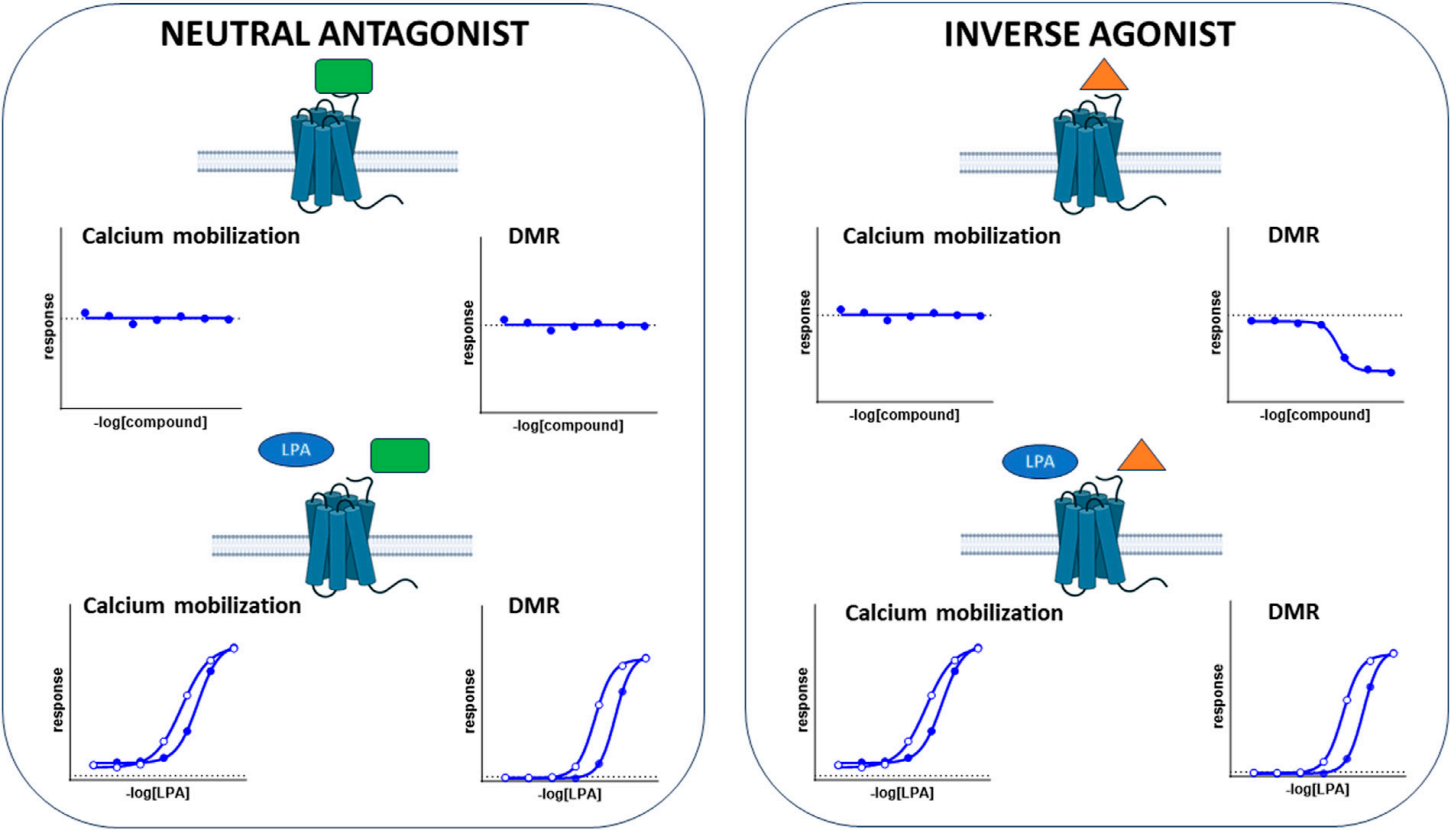
Schematic representation of the pharmacodynamic behavior of a typical LPA receptor agonist, neutral antagonist, and inverse agonist, assessed in calcium mobilization and DMR assays.

In DMR experiments, in CHO_LPA1_ cells, KI 16425, RO 6842262, and BMS-986020 not only shifted the concentration–response curve to LPA to the right, but also produced a concentration-dependent reduction in the baseline. Similarly, compound 20 produced a concentration-dependent reduction in the baseline in CHO_LPA2_ cells. Importantly, in parallel experiments performed as a control in CHO wild-type cells, all these compounds were found to be inactive, suggesting that the compounds evoked a negative DMR signal through LPA_1_ or LPA_2_ receptors, i.e., inverse agonism. However, it should be taken into account that this is not the only possible interpretation. In fact, considering that LPA receptors couple to different G proteins, similar results could be evoked by biased LPA agonists, as demonstrated in previous studies performed on the muscarinic M_2_ receptor (Bock et al., 2012). However, although we cannot rule out this possibility, we consider this second hypothesis unlikely, especially for KI 16425. In fact, a slight degree of inverse agonism activity has already been reported for this compound in GTPγS binding assay (Ohta et al., 2003) and in cAMP assay (Shimizu and Nakayama, 2017), although this has not been replicated in the inositol phosphate test (Ohta et al., 2003). Of note, differences among assays and preparations in the ability to detect inverse agonism activity are expected, since it is reported that different experimental conditions may facilitate or hamper the detection of constitutive activity of GPCRs (i.e., differences in receptor expression level, receptor desensitization, receptor/G-protein coupling, and stoichiometry) (Seifert and Wenzel-Seifert, 2002). As expected, the pEC_50_ values for the compounds as inverse agonists were close to their pA_2_/pK_B_ values obtained in antagonism studies. This result demonstrated that DMR, but not calcium mobilization assay, can be successfully used for the detection and characterization of LPA_1_ and LPA_2_ inverse agonists, and that some compounds previously classified as LPA_1_ or LPA_2_ antagonists are actually inverse agonists. It is worth mentioning that some compounds investigated in our laboratories using DMR assay (data not shown for intellectual property reasons) behaved as LPA_1_ neutral antagonists without producing *per se* a DMR negative signal, thus demonstrating the ability of this assay to discriminate between neutral antagonist and inverse agonist ligands. Figure 10 schematizes the typical results obtained with calcium mobilization and DMR assays in the presence of a neutral antagonist or an inverse agonist. The constitutive active state of LPA receptors is also indirectly suggested by results obtained with ATP. In fact, the DMR response to ATP was significantly higher in CHO wild type than in CHO cells expressing LPA receptors. This finding can be interpreted under the assumption that the constitutive activation of LPA receptors generated a higher DMR baseline, concealing the stimulatory effect of ATP. Little information is available in the literature about the possible physiological/pathological role of LPA_1_ constitutive activity and the therapeutic potential of LPA_1_ inverse agonists. There is evidence suggesting that the presence of constitutively active LPA_1_ in neuroblastoma cells may worsen the progression of cancer cells (Kato et al., 2012). Thus, it can be speculated that LPA_1_ inverse agonists may show enhanced therapeutic effectiveness as anti-cancer agents compared to pure LPA_1_ antagonists, specifically for those tumors in which LPA_1_ receptors are constitutively active. Additionally, the LPA_1_ inverse agonists identified in the frame of this study and the DMR assay may represent innovative pharmacological tools that would be useful in investigating the presence of constitutively active LPA_1_ in tissues, under both physiological and pathological conditions. This research activity may potentially be of high impact in better understanding the etiopathogenesis of the several diseases in which LPA_1_ is involved, including fibrotic diseases, and in envisioning the therapeutic potential of LPA_1_ inverse agonists vs. neutral antagonists (Berg and Clarke, 2018).

### 4.3 Compound CHI

Compound CHI behaved as an LPA_1_ inverse agonist, showing lower potency and efficacy than the standard ligands used in this study. A similar low potency value was obtained in the antagonism protocol. At the concentration used, compound CHI was found to be inactive at LPA_2_ as an antagonist and inverse agonist. However, compound CHI is a structurally novel compound and may represent the starting point for the investigation of a series of potentially more potent analogs.

In conclusion, we have demonstrated that DMR assay can be successfully used to identify and pharmacologically characterize LPA_1_ and LPA_2_ ligands. Compared to the classical and widely used calcium mobilization assay, DMR offers some advantages: in agonism experiments, it provides a more complete view of all the intracellular events subsequent to the receptor activation and allows for the identification and study of compounds acting as inverse agonists. On the other hand, in antagonism experiments, it enables the system to reach equilibrium, thus making possible the precise identification of the type of antagonism (Figure 10). Finally, in the frame of this study, compound CHI has been identified as a novel moderate-potency LPA_1_ inverse agonist.

## Data Availability

The raw data supporting the conclusions of this article will be made available by the authors, without undue reservation.
