# Supplementary figures and images for: In vitro pharmacological characterization of standard and new lysophosphatidic acid receptor antagonists using dynamic mass redistribution assay
Source: Front Pharmacol. 2023 Nov 14;14:1267414. doi: 10.3389/fphar.2023.1267414 (PMC10682101; doi:10.3389/fphar.2023.1267414)

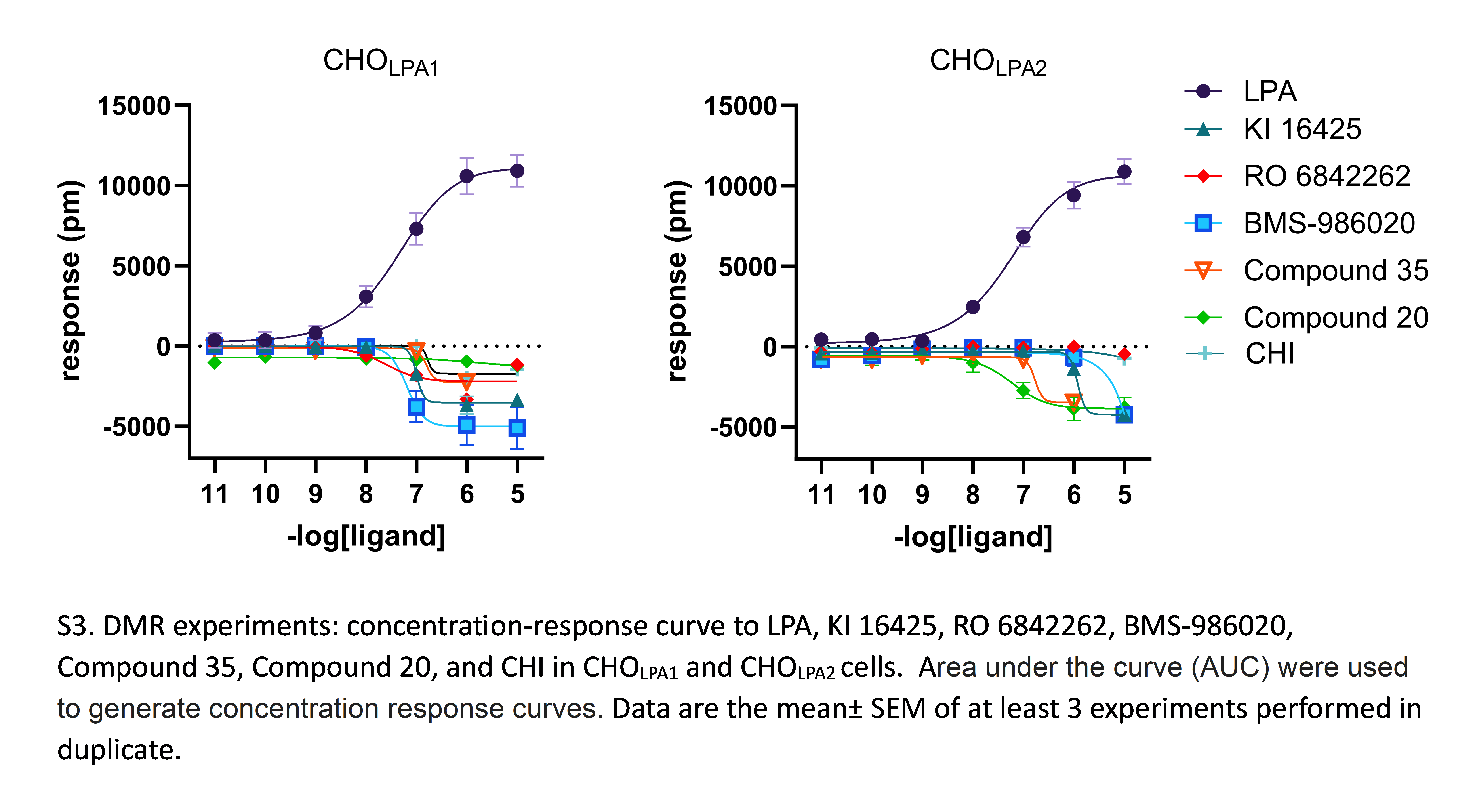

Supplement: Supplementary file 1 [file Image3.TIF]

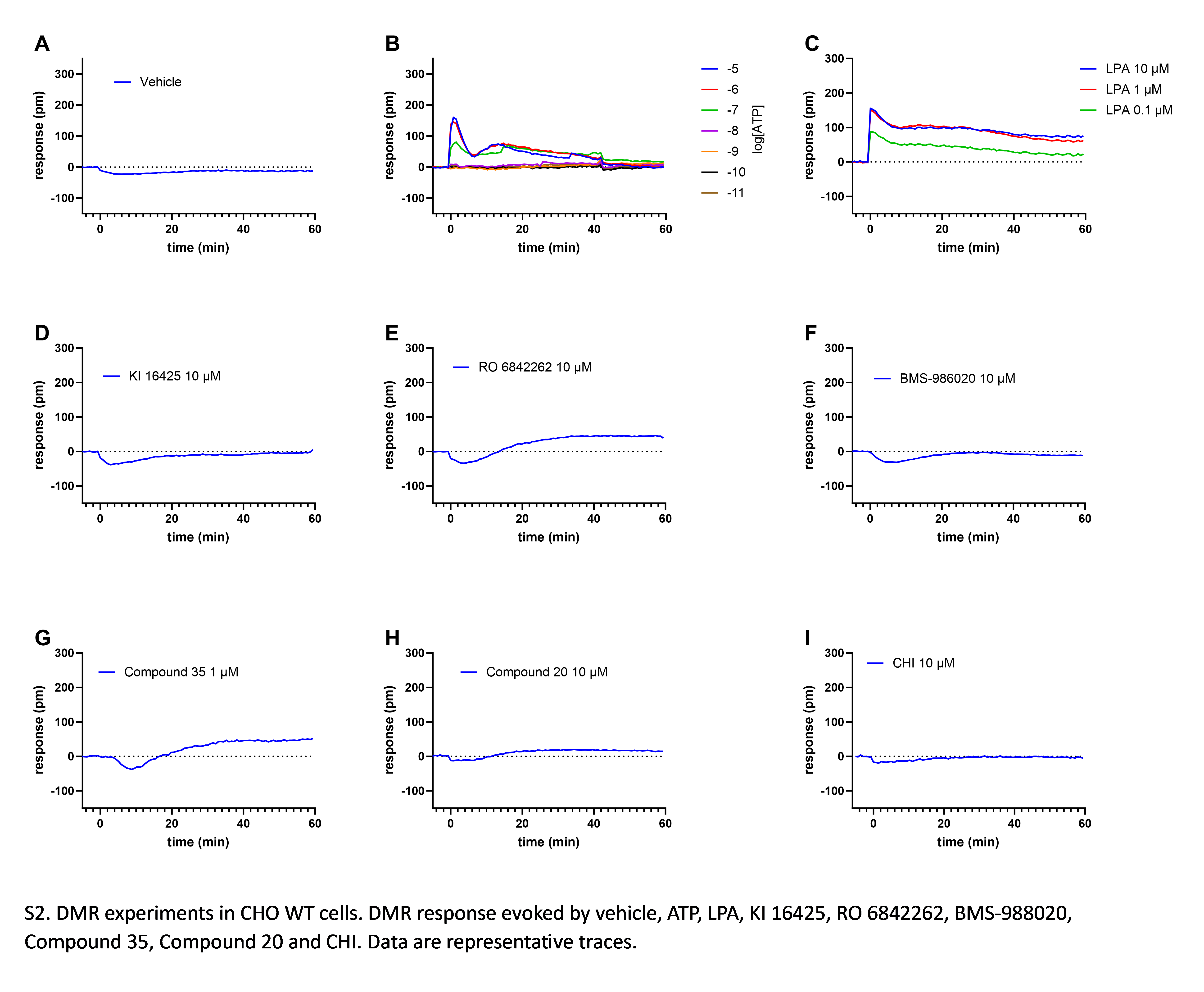

Supplement: Supplementary file 2 [file Image2.TIF]

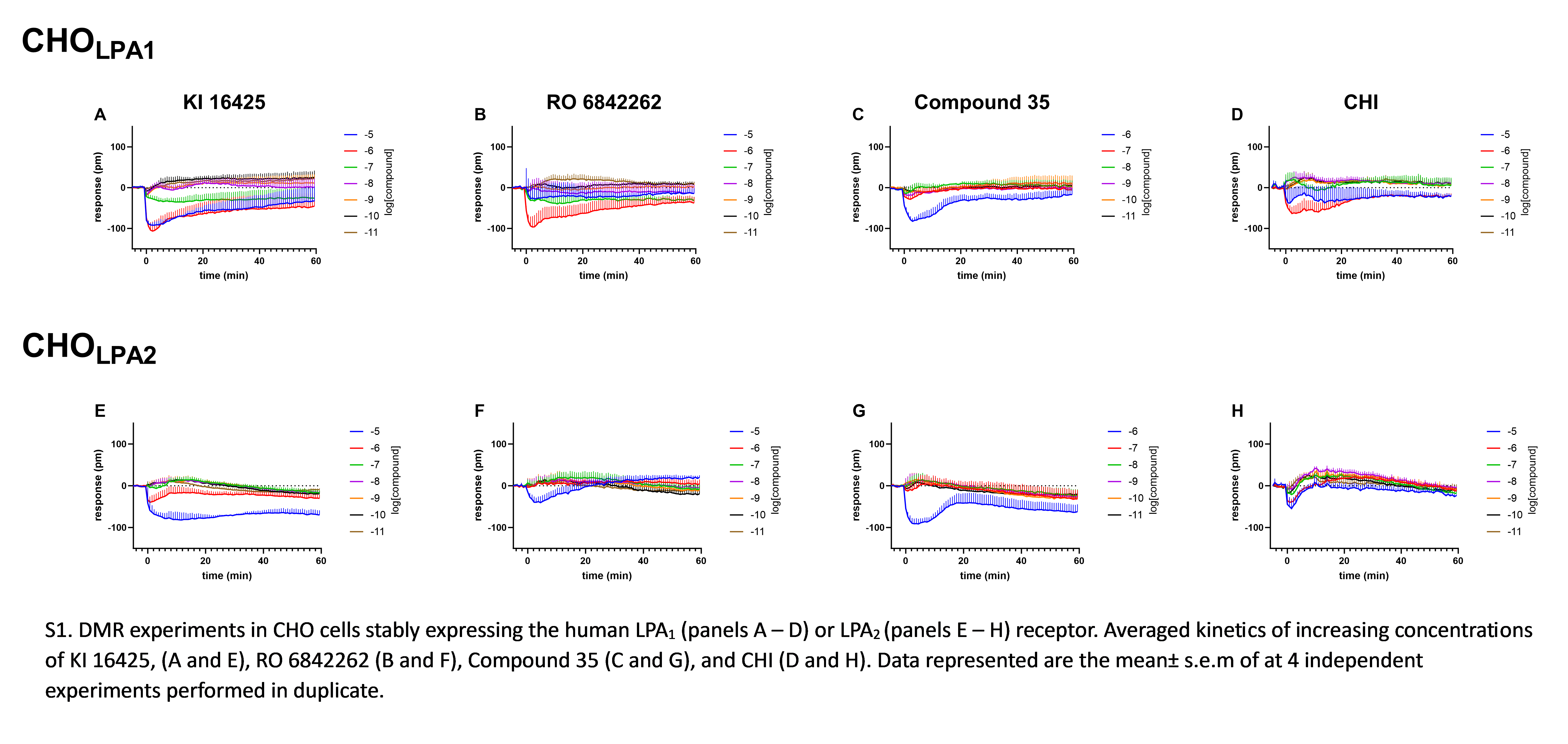

Supplement: Supplementary file 3 [file Image1.TIF]
